# A Case Report of Severe Rhabdomyolysis Following Parathyroidectomy: A Likely Synergistic Effect of Statins and General Anaesthetics

**DOI:** 10.7759/cureus.89047

**Published:** 2025-07-30

**Authors:** Mojeed Adiat, Aparna Ravikumar, Shivangi Yadav, Riya Raphael, Samraiz Nafees, Kailas Nair, Muhammad Tahir Chohan

**Affiliations:** 1 Endocrinology, York and Scarborough Teaching Hospitals NHS Foundation Trust, Scarborough, GBR; 2 Internal Medicine, York and Scarborough Teaching Hospitals NHS Foundation Trust, Scarborough, GBR; 3 Accident and Emergency, York and Scarborough Teaching Hospitals NHS Foundation Trust, Scarborough, GBR; 4 Endocrinology, Diabetes, and Metabolism, York and Scarborough Teaching Hospitals NHS Foundation Trust, Scarborough, GBR

**Keywords:** endocrinology, endocrinology and diabetes, ent surgeries, general anaesthesia, surgery

## Abstract

Rhabdomyolysis is caused by the disintegration of skeletal muscle fibres, leading to the release of toxic intracellular components into the systemic circulation resulting from direct or indirect injury to skeletal muscle, and has potential life-threatening complications such as acute renal failure. Drug-induced rhabdomyolysis, a significant subset of this syndrome, is often idiosyncratic in nature, making it challenging to study and predict.

Our report explores a case of severe rhabdomyolysis following uncomplicated general anaesthesia for parathyroidectomy in a patient who was on statin therapy perioperatively for 14 years and had relatively no side effects arising from it.

## Introduction

Rhabdomyolysis is characterised by accelerated skeletal muscle fibre destruction [1]. This syndrome encompasses diverse precipitating factors, including mechanical trauma, tissue hypoxia, infectious processes, hereditary defects, therapeutic medications, and environmental toxins [2]. The underlying pathophysiology involves the disruption of muscle cell membranes, leading to the systemic release of intracellular components, including creatine kinase (CK), myoglobin, potassium, and phosphate [2,3]. The clinical spectrum ranges from subclinical enzyme elevation to life-threatening complications such as renal failure, metabolic derangements, and coagulopathy [2,4].

Non-traumatic rhabdomyolysis encompasses a diverse array of precipitating mechanisms [5]. Drug-related pathways constitute a significant proportion of iatrogenic presentations, with particular emphasis on hydroxymethylglutaryl-CoA reductase inhibitors (statins), anaesthetic compounds including propofol, perioperative pharmacological agents, and prolonged immobilisation [2,5,6].

Hydroxymethylglutaryl-CoA reductase inhibitors are the main type of medication used to lower cholesterol and are essential for preventing and treating atherosclerotic cardiovascular disease [7-9]. While demonstrating substantial cardiovascular benefits, these agents exhibit myotoxic potential ranging from subclinical myalgias to fulminant muscle necrosis. Muscle-related adverse reactions occur in approximately 10-25% of treated patients [9,10].

Anaesthetic compounds, including depolarising neuromuscular blocking agents, volatile halogenated gases, and propofol, demonstrate established associations with muscle breakdown in vulnerable populations. Individuals harbouring inherited neuromuscular pathology or mitochondrial dysfunction demonstrate a heightened susceptibility to rhabdomyolysis [2,5].

Establishing a definitive causal link between specific medications and rhabdomyolysis remains diagnostically complex [6,8]. Therefore, causality is inferred primarily through temporal association, clinical context, and the systematic exclusion of other plausible aetiologies [6,8,11].

We describe a patient who developed severe rhabdomyolysis 14 days after routine parathyroidectomy. Following a comprehensive evaluation excluding alternative aetiologies, the presentation was attributed to potential synergistic effects between concurrent statin therapy and intraoperative volatile anaesthetic exposure.

## Case presentation

A 75-year-old woman presented with generalised myopathy, progressive weakness, and leg pain that predominantly affected the proximal muscles for the past 10 days. Physical examination revealed signs of proximal myopathy without other features suggestive of endocrinopathy. Her medical history was significant for ischaemic heart disease, familial hypercholesterolaemia, prediabetes, stage IIIa chronic kidney disease, osteoporosis, and primary hyperparathyroidism. She had been on a stable medication regimen including aspirin, ramipril, ezetimibe, and rosuvastatin (40 mg daily) for 15 years, with no prior evidence of hepatotoxicity or myotoxicity.

Fourteen days before the presentation, she underwent an uncomplicated parathyroidectomy for primary hyperparathyroidism. The procedure lasted 45 minutes, and no intraoperative complications were noted. Anaesthesia was induced and maintained with propofol and sevoflurane. The postoperative recovery was uneventful, and she was discharged with instructions to follow up in the endocrine clinic. However, she developed generalised myalgia beginning four days after surgery, which progressively worsened and was accompanied by weakness and muscle pain, prompting a hospital readmission.

There was no history of trauma, prolonged immobility, intense physical exertion, seizures, or electrical injury. Furthermore, she had no personal or family history of autoimmune disorders.

Initial laboratory investigations, including complete blood count, renal function tests, thyroid function tests, C-reactive protein, bone profile, coagulation studies, and lipid profile, were within normal limits. However, CK and alanine transaminase (ALT) were significantly elevated, as summarised below in Table 1. 

**Table 1:** Laboratory investigations at the time of presentation eGFR: estimated glomerular filtration rate; ALT: alanine transaminase; CRP: C-reactive protein

Laboratory investigations	Values	Normal range
Urea	6.4 mmol/L	2.5-7.8 mmol/L
Creatinine	69 µmol/L	59-104 µmol/L
eGFR	71.9 ml/min/1.73 m^2^	>60 ml/min/1.73 m^2^
Creatine kinase	27,096 U/L	25-200 U/L
Sodium	137 mmol/L	135-145 mmol/L
Potassium	4.1 mmol/L	3.5-5.5 mmol/L
Calcium	2.6 mmol/L	2.1-2.6 mmol/L
Phosphate	0.82 mmol/L	0.8-1.5 mmol/L
ALT	717 U/L	0-34 U/L
CRP	13 mg/L	<3 mg/L

A comprehensive autoantibody panel was conducted, yielding negative results, as summarised in Table 2 below.

**Table 2:** Autoantibody panel ANA: antinuclear antibodies

Antibodies	Values	Normal range
ANA	Negative	n/a
Anti-JO antibodies	Negative	n/a
Rheumatoid factor	Negative	n/a

She was diagnosed with rhabdomyolysis and managed with aggressive fluid resuscitation. Her CK levels gradually improved over the next couple of days and did not warrant haemodialysis. Following recovery, her statins were permanently discontinued. 

## Discussion

Rhabdomyolysis is typically identified by a significant elevation in serum CK, often 10-25 times above the upper reference limit, along with compatible clinical features [10]. In our patient, the marked rise in CK levels (>20,000 U/L) and the presence of severe myalgia were strongly indicative of rhabdomyolysis [4]. The elevation in ALT levels can also be explained by skeletal muscle injury, as ALT is released during muscle fibre breakdown [2].

A wide range of potential causes should be considered in cases of rhabdomyolysis. These include traumatic injuries, impaired calcium regulation in muscle tissue, as seen in malignant hyperthermia (MH), infections, autoimmune or hereditary myopathies (e.g., Becker muscular dystrophy), electrolyte imbalances like hypokalaemia or hypophosphatemia, and drug-related muscle toxicity, such as from statins [2,4,12,13].

In our case, no signs of trauma, infection, hypoxia, or prolonged immobility were found. The patient's autoimmune screening returned negative, ruling out rheumatologic causes.

There are reports in the literature of delayed-onset MH and rhabdomyolysis following the administration of volatile anaesthetic agents, such as sevoflurane, in genetically predisposed individuals [4]. Neuromuscular disorders, including muscular dystrophies, can increase the likelihood of such reactions [14]. However, these disorders typically present earlier in life, and our elderly patient had no history of neuromuscular disease or family history suggestive of such conditions [2,11,14].

It is plausible that the concurrent use of statins and exposure to general anaesthesia contributed synergistically to muscle injury in this patient [11,14]. This interaction is especially concerning in individuals undergoing surgery while on long-term statin therapy [11,14]. Although the patient had tolerated statins previously without incident, the perioperative use of anaesthetic agents may have triggered an acute episode in the absence of other contributing factors.

Given the absence of identifiable alternative causes and the clear temporal association between anaesthetic exposure and symptom onset, we postulate that the synergistic interaction between long-term statin therapy and volatile anaesthetic agents may have contributed to the development of rhabdomyolysis in this patient. Establishing a definitive causal relationship between pharmacologic exposures and rhabdomyolysis remains challenging [6,8]. The current literature lacks robust data from large-scale clinical trials, prospective cohorts, or meta-analyses that specifically evaluate the potential for synergistic myotoxicity between these commonly used agents. As such, clinical judgement plays a crucial role in causality assessment [6,8,11,14]. In this case, our conclusion was supported by the temporal proximity of events, characteristic clinical features, and the systematic exclusion of other known aetiologies.

Current perioperative guidelines generally recommend continuing statin therapy during the perioperative period, including for non-cardiac surgery, due to their cardiovascular protective effects and relatively low risk of adverse muscle events [15].

However, clinicians should carefully evaluate individual patient risk factors, such as pre-existing myopathy, renal impairment, or potential drug interactions, which may warrant temporary cessation.

Our case highlights the importance of heightened vigilance for muscle toxicity, particularly in patients on long-term statin therapy undergoing surgery, where perioperative factors may increase the risk of adverse muscle events.

## Conclusions

We present a patient who developed severe rhabdomyolysis in the postoperative period. Although a single definitive cause could not be established, the close temporal association with surgery, combined with the patient's longstanding, previously uneventful statin therapy, raises the possibility of synergistic myotoxic effects involving perioperative factors such as statins, anaesthetic exposure, and surgical stress. In the absence of other identifiable risk factors, this case highlights the need for heightened clinical awareness in the perioperative management of patients on chronic statin therapy. Decisions regarding the continuation or temporary cessation of statins should be individualised, weighing the potential risk of muscle injury against the well-established cardiovascular benefits.
